# Association of defects of enamel with polymorphisms in the vitamin D receptor and parathyroid hormone genes

**DOI:** 10.1590/0103-6440202405900

**Published:** 2024-06-24

**Authors:** Amanda Renostro-Souza, Gabriela Fonseca-Souza, Erika Calvano Küchler, Katia Regina Felizardo Vasconcelos, Juliana Feltrin-Souza, Christian Kirschneck, Mírian Aiko Nakane Matsumoto, Cesar Penazzo Lepri, Maria Angelica Hueb de Menezes Oliveira, Geraldo Thedei

**Affiliations:** 1 Department of Biomaterials, University of Uberaba, Uberaba, MG, Brazil.; 2 Department of Stomatology, Federal University of Paraná, Curitiba, PR, Brazil.; 3 Department of Pediatric Dentistry, School of Dentistry of Ribeirão Preto, University of São Paulo, Ribeirão Preto, São Paulo, Brazil; 4 Department of Orthodontics, University of Bonn, Welschnonnenstr, Bonn, Germany

**Keywords:** vitamin D, parathyroid hormone, Developmental Defects of Enamel, Polymorphism, Single Nucleotide

## Abstract

This cross-sectional study aimed to investigate the association between developmental defects of enamel (DDE) and single nucleotide polymorphisms (SNPs) in the genes encoding the vitamin D receptor (VDR) and parathyroid hormone (PTH). Orthodontic patients receiving treatment at a dental school were selected through convenience sampling. Intra-oral photographs were used to assess DDE, which were classified according to the criteria proposed by Ghanim et al. (2015) by a single calibrated examiner (Kappa>0.80). Enamel hypoplasia, molar-incisor hypomineralization (MIH), hypomimineralized second primary molar (HSPM), and non-MIH/HSPM demarcated opacities were considered for the analysis. Genomic DNA was extracted from buccal cells. The SNPs in VDR (rs7975232) and PHT (rs694, rs6256, and rs307247) were genotyped using real-time polymerase chain reactions (PCR). Statistical analyses were performed using the PLINK software (version 1.03, designed by Shaun Purcell, EUA). Chi-square or Fisher's exact tests were performed at a significance level of 5%. Ninety-one (n=91) patients (49 females and 42 males) (mean age of 14.1±5.8 years) were included. The frequency of DDE was 38.5% (35 patients). Genotype distributions were in Hardy-Weinberg equilibrium. No significant statistical association was found between DDE and the SNPs evaluated. A borderline association (p=0.09) was observed between DDE and the CC haplotype for SNP rs7975232 in VDR. In conclusion, the selected SNPs in VDR and PTH genes were not associated with DDE in the studied samples.

## Resumo

Este estudo transversal teve como objetivo investigar a associação entre defeitos de desenvolvimento do esmalte (DDE) e polimorfismos de nucleotídeo único (SNPs) nos genes que codificam o receptor da vitamina D (VDR) e o hormônio da paratireoide (PTH). Pacientes ortodônticos em tratamento em uma escola Odontologia foram selecionados por amostragem de conveniência. Os DDEs foram avaliados e classificados por um examinador calibrado (Kappa>0,80) através de fotografias intraorais de acordo com os critérios propostos por Ghanim et al. (2015). Os tipos de DDE considerados para análise foram: hipoplasia de esmalte, hipomineralização molar-incisivo (HMI), hipomineralização de segundos molares decíduos (HSMD) e opacidades demarcadas não-HMI/HSMD. O DNA gnômico foi extraído de células bucais. Os SNPs em *VDR* (rs7975232) e *PTH* (rs694, rs6256 e rs307247) foram genotipados por PCR em tempo real. As análises estatísticas foram realizadas utilizando o software PLINK (versão 1.03, concebido por Shaun Purcell, EUA). Foram feitos teste de qui-quadrado e teste exato de Fisher com um nível de significância de 5%. Foram incluídos noventa e um (n=91) pacientes (49 do sexo feminino e 42 do sexo masculino) (idade média de 14,1±5,8 anos). A frequência de DDE foi de 38,5% (35 pacientes). As distribuições genotípicas estavam em equilíbrio de Hardy-Weinberg. Não foi encontrada associação estatisticamente significante entre os DDEs e os SNPs avaliados. Foi observada uma associação limítrofe (p=0,09) entre a DDE e o haplótipo CC para o SNP rs7975232 no *VDR*. Em conclusão, os SNPs seleccionados nos genes *VDR* e *PTH* não foram associados à DDE nas amostras estudadas.

## Introduction

The dental enamel formation, known as amelogenesis, is a complex process regulated by genes and influenced by genetic and environmental factors ^1^. Disturbances during this process may lead to developmental defects of enamel (DDE), such as hypoplasia and hypomineralizations ^2^. Hypoplasia is a quantitative defect associated with a reduced localized thickness of enamel ^2^ ^,^ ^3^. Hypomineralizations are qualitative defects characterized by a reduced mineral content, visualized as alterations in the enamel translucency ^4^.

Clinically, hypomineralizations are divided into diffuse and demarcated opacities. Diffuse opacities are observed in cases of dental fluorosis, presenting a linear, patchy, or confluent distribution with no clear boundary with the adjacent normal enamel ^2^ ^,^ ^3^. On the other hand, demarcated opacities have a clearly defined boundary from adjacent enamel. Ghanim et al. ^3^ divided this type of opacity into two groups: molar incisor-hypomineralization/hypomineralization of second primary molars (MIH/HSPM) and non-MIH/HSPM demarcated opacities. MIH and HSPM affect one or more first permanent molars, with or without involvement of incisors and second primary molars, respectively. Non-MIH/ HSPM opacities affect primary or permanent teeth other than MIH/HSPM ^3^.

The etiology of DDE is complex, combining environmental and genetic factors ^4^ ^,^ ^5^. It is known that dental fluorosis is influenced by genetic factors but depends on the excessive ingestion of fluoride during amelogenesis to occurs ^6^. However, the causes of other DDEs, such as MIH/HSPM and hypoplasia, are still unclear. Several expositions during the prenatal, perinatal, and postnatal periods, as well as genetic factors, have been pointed to disrupt amelogenesis pathways, leading to these DDEs ^4^ ^,^ ^5^.

The metabolism of calcium homeostasis is an important amelogenesis pathway ^5^. Calcium is the main ionic species of the mineralized enamel matrix ^7^. Besides that, this ion regulates the expression of enamel proteins ^7^ and plays a role in ameloblast cell differentiation ^8^. Calcium homeostasis is related to the levels of vitamin D, which exerts its function by binding to the nuclear vitamin D receptor (VDR), and parathormone (PTH) ^5^. These factors form a tightly controlled feedback cycle: PTH stimulates vitamin D synthesis in the kidney, while vitamin D exerts negative feedback on PTH secretion ^9^.

Although VDR and PTH have an essential role in calcium homeostasis and seem to be expressed during dental development ^10^ ^,^ ^11^ ^,^ ^12^, the evidence about the association between DDE and these genes is scarce. Animal studies observed that alterations in VDR may result in impaired enamel development ^13^ ^,^ ^14^. In humans, two studies were conducted aiming to investigate the association between MIH and single nucleotide polymorphisms (SNPs) in VDR ^15^ ^,^ ^16^, finding a significant association between this type of DDE and rs739837 ^15^ and rs78783628 ^16^. However, no research has already been conducted to evaluate the role of SNPs in this gene in other types of DDE. The knowledge about PTH is even more limited. Evidence from animal studies suggests that reduced levels of PTH induce hypocalcemia and consequently enamel alterations ^17^.

Considering the potential role of VDR and PTH in amelogenesis, as well as the limited evidence about the association between DDE and these genes, this cross-sectional study aimed to investigate the association between DDE (excluding dental fluorosis) and SNPs in the genes encoding VDR and PTH.

## Methods

### Sample

This cross-sectional study was approved by the local Ethics Committee (number: 50765715.3.0000.5419). Informed consent was obtained from all patients or their legal guardians in the case of patients under 18 years. This study was carried out following the Strengthening the Reporting of Genetic Association Studies (STREGA) ^18^.

Orthodontic patients (children and adults) receiving treatment at the University of São Paulo (Ribeirão Preto, São Paulo, Brazil) were selected through a convenience sampling. Healthy unrelated patients with dental records, good-quality photographs, and sufficient DNA samples were included in this study. Patients who presented dental fluorosis, dentinogenesis imperfecta, amelogenesis imperfecta, with syndromes, oral clefts, unhealthy systemic conditions, endocrinal problems, and undergoing hormonal treatment were excluded.

### Phenotypes definition

**Figure 1:**
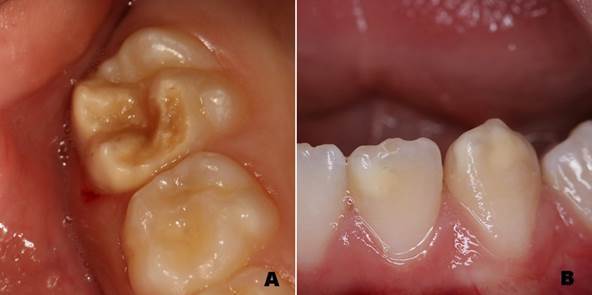
A. First permanent molar affected by MIH; B. Lower incisor and canine with demarcated opacities.

Standardized intra-oral photographs from orthodontic records were used to assess DDE (Figure 1). Five intra-oral photographs from each patient (frontal, right, left, maxillary, and mandibular occlusal views) were taken using a digital camera, artificial lighting, a mouth retractor, and dental photography mirrors.

One examiner was trained and calibrated (kappa> 0.8) to diagnose DDE using the criteria proposed by Ghanim et al. ^3^: 0 - no visible enamel defect; 11 - diffuse opacities (dental fluorosis); 12 - hypoplasia; 13 - amelogenesis imperfecta; 14 - non-MIH/HSPM demarcated opacities; 21 - White or creamy opacities; 22 - yellow or brown opacities; 3 - Post-eruptive breakdown; 4 - atypical restorations; 5 - atypical caries; 6 - atypical extraction. Codes 21, 22, and 3 to 6 are used to classify only MIH/HSPM according to the severity degree.

Once dental fluorosis and amelogenesis imperfecta have different etiological backgrounds from the other DDE, patients presenting one of these types of defects were excluded. Thus, the "DDE” phenotype was defined as patients with at least one tooth coded as 12, 14, 21, 22, 3, 4, 5, and 6. The “non-DDE” was defined as patients without DDE coded as 0.

### Dna extraction and genotyping

Buccal cells for genomic analysis were collected by rinsing the mouth for one minute with 5 mL of saline solution. The saliva samples were stored at -20°C until DNA extraction. Genomic DNA for genotype analysis was extracted from buccal cells following the method described by Küchler et al*.* ^19^. DNA concentration and purity were determined by spectrophotometry using NanoDrop 2000 (Thermo Fisher Scientific, Waltham, MA, USA) and diluted to 4 ng/mL.

Genotyping was performed using real-time polymerase chain reactions (PCR) (Applied Biosystems, StepOnePlus Real-Time PCR System, Thermo Fisher Scientific, Foster City, CA, USA). Four SNPs were selected (rs7975232 in *VDR* and rs694, rs6256, and rs307247 in *PTH*) based on their minor allele frequency (which should be higher than 30%), and linkage disequilibrium. These well-investigated SNPs present a possible clinical impact in VDR ^20^ ^,^ ^21^ ^,^ ^22^ ^)^ and are associated with alterations in PTH serum levels ^23^ ^,^ ^24^ ^,^ ^25^ ^,^ ^26^. The characteristics of the selected SNPs are expressed in Table 1.

**Table 1:** Characteristics of the selected single nucleotide polymorphisms.

SNP	rs7975232	rs694	rs6256	rs307247
Gene	*VDR*	*PTH*	*PTH*	*PTH*
Position	47845054	13492870	13492506	13491931
Minor Allele Frequency	0.45	0.51	0.12	0.39
Base change	C>A	C>T	G>A, T	G > A
Function	Intron Variant	Intron variant	Stop gained	500B Downstream Variant

Obtained from: https://www.ncbi.nlm.nih.gov/snp/

### Statistical analysis

For the statistical analysis, the phenotypes were categorized as "DDE" and "non-DDE". Hardy-Weinberg equilibrium was assessed for each SNP using the chi-square test, considering the critical value of 3.84. Chi-square or Fisher's exact tests, with odds ratio calculations and their respective 95% confidence intervals, were used to compare genotype and allele distributions between the phenotypes in the co-dominant, dominant, and recessive models. A significance level (alpha) of 5% was established for all comparisons. The data analyses were carried out using the PLINK software (version 1.03, designed by Shaun Purcell, EUA).

## Results

**Figure 2:**
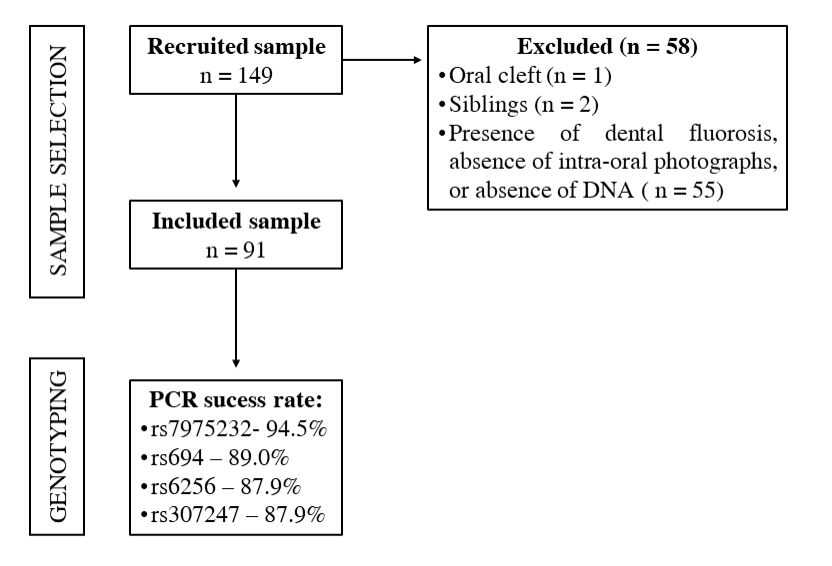
Flow chart of the study sample selection and genotyping.

A total of 149 patients were initially recruited to participate in this study. Of these, fifty-eight were excluded due to the following reasons: related patients (siblings), presence of oral cleft, absence of intra-oral photographs, presence of dental fluorosis, and insufficient DNA samples. The final sample included 91 patients (49 females and 42 males) aged between 9 and 40 years (mean age=14.1± 5.8). The flowchart of the study sample selection is presented in Figure 2.

DDE was observed in 35 patients (38.5%): 3 patients presented enamel hypoplasia, 15 MIH/HSPM, 13 non-MIH/HSPM demarcated opacities, and 4 had both MIH/HSPM and non-MIH/HSPM demarcated opacities. A total of 56 (61.5%) patients did not present any enamel defect and were used as controls (Table 2).

Genotyping was performed for all patients included (n=91). The success rate of the PCR is shown in Figure 2 according to the analyzed SNP. All SNPs evaluated were in Hardy-Weinberg equilibrium (chi-square < 3.84) (Table 3). No significant statistical significance was found between DDE and the SNPs evaluated in any of the models (co-dominant, dominant, and recessive models) (p>0.05). However, a borderline association (p=0.09) was observed between DDE and the CC haplotype for SNP rs7975232 in VDR (Table 4).

**Table 2:** Prevalence of DDE in the study population (n=91).

Type of defect	Yes n (%)
Enamel hypoplasia	3 (3.3%)
MIH/HSPM	19 (20.9)
Non-MIH/HSPM hypomineralization	16 (17.6)

Abbreviations: MIH - Molar Incisor Hypomineralization; HSPM - Hypomineralized Second Primary Molars.

**Table 3:** Handy-Weinberg equilibrium and the distribution of observed frequency of the genotype on the study population.

Gene	Polymorphism	Genotype	n (%)	Hardy-Weinberg Equilibrium Chi-square
*VDR*	rs7975232	AA	39 (45.3)	0.1548
		AC	39 (45.3)	
		CC	8 (9.4)	
*PTH*	rs694	CC	12 (14.8)	1.4992
		CT	45 (55.5)	
		TT	24 (29.7)	
	rs6256	GG	64 (80.0)	0.013
		GT	15 (18.8)	
		TT	1 (1.2)	
	rs307247	AA	11 (13.8)	2.1864
		AG	29 (36.2)	
		GG	40 (50.0)	

**Table 4:** Evaluation of the SNP according to the groups in the codominant, dominant, and recessive models.

SNP (gene)	Phenotype	Genotype frequencies n (%)			**Association test *P* values**		
					Codominant model	Dominant model	Recessive model
rs7975232 (*VDR*)		AA	AC	CC	AA vs. AC vs. CC	AA+AC vs. CC	AA vs. AC+CC
	DDE	16 (45.7)	13 (37.1)	6 (17.1)	0.092	0.090	>0.999
	Non-DDE	23 (45.1)	26 (51.0)	2 (3.9)			
rs694 (*PTH*)		CC	CT	TT	CC vs. CT vs. TT	CC+CT vs. TT	CC vs. CT+TT
	DDE	5 (15.2)	19 (57.6)	9 (27.3)	0.928	0.891	>0.999
	Non-DDE	7 (14.6)	26 (54.2)	15 (31.3)			
rs6256 (*PTH*)		GG	GT	TT	GG vs. GT vs. TT	GG+GT vs. TT	GG vs. GT+TT
	DDE	24 (72.7)	8 (24.2)	1 (3.0)	0.259	0.858	0.281
	Non-DDE	40 (85.1)	7 (14.9)	0 (0.0)			
rs307247 (*PTH*)		AA	AG	GG	AA vs. AG vs. GG	AA+AG vs. GG	AA vs. AG+GG
	DDE	5 (15.2)	11 (33.3)	17 (51.5)	0.888	>0.999	>0.999
	Non-DDE	6 (12.8)	18 (38.3)	23 (48.9)			

Note: The total number for each SNP change according to the success PCR rate. Samples that were not amplified were not included in the analysis.

## Discussion

In this study, enamel hypoplasia, MIH/HSPM, and non-MIH/HSPM demarcated opacities were grouped as the main phenotype “DDE” once the hypothesis raised here is that the candidate genes *VDR* and *PTH* are involved in DDE regardless of the subphenotype. Cases of dental fluorosis and amelogenesis imperfecta were not included since they present a different etiological background from the other DDEs. Although genetic factors influence dental fluorosis susceptibility, it is known that the excessive ingestion of fluoride during tooth development is necessary for its occurrence ^6^ ^,^ ^27^ ^,^ ^28^. *Amelogenesis imperfecta* consists of a heterogeneous group of genetic conditions characterized by defects in the formation of enamel in all teeth in both primary and permanent dentitions ^29^.

DDE has been associated with SNPs in genes involved in enamel development ^30^ ^,^ ^31^, immune response ^31^, and estrogen signaling pathway ^32^. However, few studies investigated the association between DDE and SNPs in genes related to calcium homeostasis ^15^ ^,^ ^16^. Thus, in our study, we evaluated if SNPs of genes encoding VDR and PTH are implicated in the risk of developing DDE.

Disruptions in calcium homeostasis may lead to alterations in enamel development ^33^ *. VDR* is a nuclear transcription factor that mediates the actions of the active form of vitamin D *(1,25-*dihydroxyvitamin *D - 1,25(OH)2D).* Besides playing an important role in the regulation of serum calcium levels, VDR is expressed in cells directly involved in mineralized tissue formation, including the ameloblasts ^*34*^ *.* In the present study, a borderline association (p=0.09) was observed between DDE and the CC haplotype for the SNP rs7975232 in VDR. Previous research found a significant association between MIH and the SNPs *rs739837* ^*15*^ and rs78783628 ^16^ in VDR, reinforcing the possible role of this gene in the occurrence of DDE.

Parathyroid hormone (PTH) is an endocrine factor secreted by the parathyroid gland ^11^. In vitro studies have provided evidence that this factor has some influence on odontogenesis (^11^, 35, ^3^). Additionally, a study in rats ^17^ investigated the effects of a thyroid-parathyroidectomy on enamel development and observed that the intervention induced severe hypocalcemia, affecting the enamel shape and mineralization, which suggests that ameloblasts may be sensitive to PTH. Despite that, no association between DDE and the four analyzed SNPs in *PTH* were found in our study.

Although in the present study, we did not find an association between DDE and the analyzed SNPs in *VDR* and *PTH* genes, we emphasize that this result should be interpreted with caution. In our analysis, the types of DDE were not stratified due to the small sample size, which comprised only 35 patients with DDE. In order to confirm if the SNP rs7975232 in *the VDR* gene and the SNPs rs307247, rs694, and rs6256 in *PTH* genes are involved in these defects, a larger sample is necessary. Another relevant limitation to be highlighted here is related to the fact that DDE presents a complex etiology involving gene and environmental interactions, and we did not consider the relationship between these factors. Besides that, in our analysis, only a few SNPs were explored. *VDR* is a gene with some well-known SNPs. It is possible that other SNPs in *VDR*, or also in *PTH*, as well as their interaction, could be involved in DDEs. Therefore, we suggest new studies involving a larger sample and the evaluation of a broader range of SNPs, especially in *VDR*.

## Conclusions

In the present study, SNPs in VDR and PTH were not associated with the etiology of human DDE.
